# Wound care counseling of patients with hidradenitis suppurativa: perspectives of dermatologists

**DOI:** 10.1097/JW9.0000000000000096

**Published:** 2023-07-18

**Authors:** Sneha Poondru, Kourtney Scott, Julia M. Riley

**Affiliations:** a Department of Dermatology, Northwestern University Feinberg School of Medicine, Chicago, Illinois

**Keywords:** clothing modifications, dressings, flares, hidradenitis suppurativa, wound care

**What is known about this subject in regard to women and their families?**

- Hidradenitis suppurativa (HS) is a chronic, debilitating skin condition that disproportionately affects women, Black individuals, and those of low socioeconomic status.
- As HS affects intertriginous areas, such as the groin, axillae, and inframammary folds, wound care of acute flares and chronic, draining wounds can be challenging and is an unmet need.

**What is new frosm this article as messages for women and their families?**

- Over one-third of surveyed dermatologists do not counsel patients with HS about wound care dressings, more than half do not discuss clothing modifications, and only half reported offering insurance-covered wound care supplies for patients.
- Patient education on at-home wound care management, including options for dressings and topical agents for HS flares, needs to be improved.
- Clothing modifications—such as wearing “boy shorts” or loose boxers, instead of tight briefs, and wireless bras with thick bands—must be discussed with women with HS.

Wound care in hidradenitis suppurativa (HS) is an unmet need and consists of management of both acute flares and chronic wounds with daily drainage.^1^ This study evaluates wound care counseling and product recommendations dermatologists offer to patients with HS.

An anonymous, multiple-choice, online questionnaire (Supplementary Figure S1, http://links.lww.com/IJWD/A21) was distributed to currently-practicing, board-certified dermatologists in the United States via an email listserv between August and October 2022. The χ^2^ and Fisher’s exact tests were performed with Microsoft Excel version 16.65. The Northwestern University Institutional Review Board approved this study.

In total, 74 dermatologists accessed the questionnaire, and 50 completed it (67.6%). Thirty-eight percent (n = 19) self-identified as HS experts (Table 1). Regarding counseling to HS patients, 62% (n = 31) of dermatologists discuss wound care dressings, 74% (n = 37) discuss at-home management of acute flares, and 48% (n = 24) discuss clothing modifications. Overall, 70% (n = 35) believed that patients with HS are not well-educated about wound care practices, and 90% (n = 45) agreed that patients need more wound care education.

**Table 1:** Characteristics of dermatologists in the sample

Characteristic	No. (%)
Total	50
Years in practice	
<5 y	16 (32)
5-10 y	13 (26)
10-15 y	13 (26)
15+ y	8 (16)
Location of practice	
Northeast	8 (16)
Midwest	22 (44)
South	9 (18)
West	11 (22)
Setting of practice	
Urban	23 (46)
Suburban	21 (42)
Rural	6 (12)
HS expert	
Yes	19 (38)
No	31 (62)
Procedures performed for HS	
Intralesional steroid	48 (96)
Deroofing	28 (56)
Local excision	25 (50)
Laser hair removal	20 (40)
Ablative laser	3 (6)
Counseling topics discussed	
Wound care dressings	31 (62)
Management of acute flares	37 (74)
Clothing modifications	24 (48)

HS, hidradenitis suppurativa; y, years.

Commonly recommended dressings were abdominal pads (80%; n = 40), gauze (76%; n = 38), and panty liners or menstrual pads (52%; n = 26). Half of the respondents (n = 25) reported ordering insurance-covered wound care supplies for patients with HS. For at-home management of flares, warm compresses (76%; n = 38), bleach baths (50%; n = 25), zinc oxide cream (28%; n = 14), Epsom salt baths (18%; n = 9), and Vicks VapoRub (12%; n = 6) were recommended (Fig. 1A). Warm compresses, followed by bleach baths and Epsom salt baths, had the highest proportion of dermatologists rating the product efficacy as good or excellent for flares. However, for the remaining products, most respondents were unsure about their efficacy (Fig. 1B). Clothing modifications recommended included “boy shorts” or loose boxers for underwear (48%; n = 24), wireless bras (34%; n = 17), seamless pants (20%; n = 10), bra liners (16%; n = 8), and HidraWear products (10%; n = 5). Dermatologists who identified as HS experts were more likely than nonexperts to discuss Vicks VapoRub (31% vs 3%; *P* < .01), abdominal pads (90% vs 72%; *P* < .01) and panty liners (79% vs 36%; *P* < .01), and clothing modifications (74% vs 32%; *P* < .01).

**Fig. 1.**
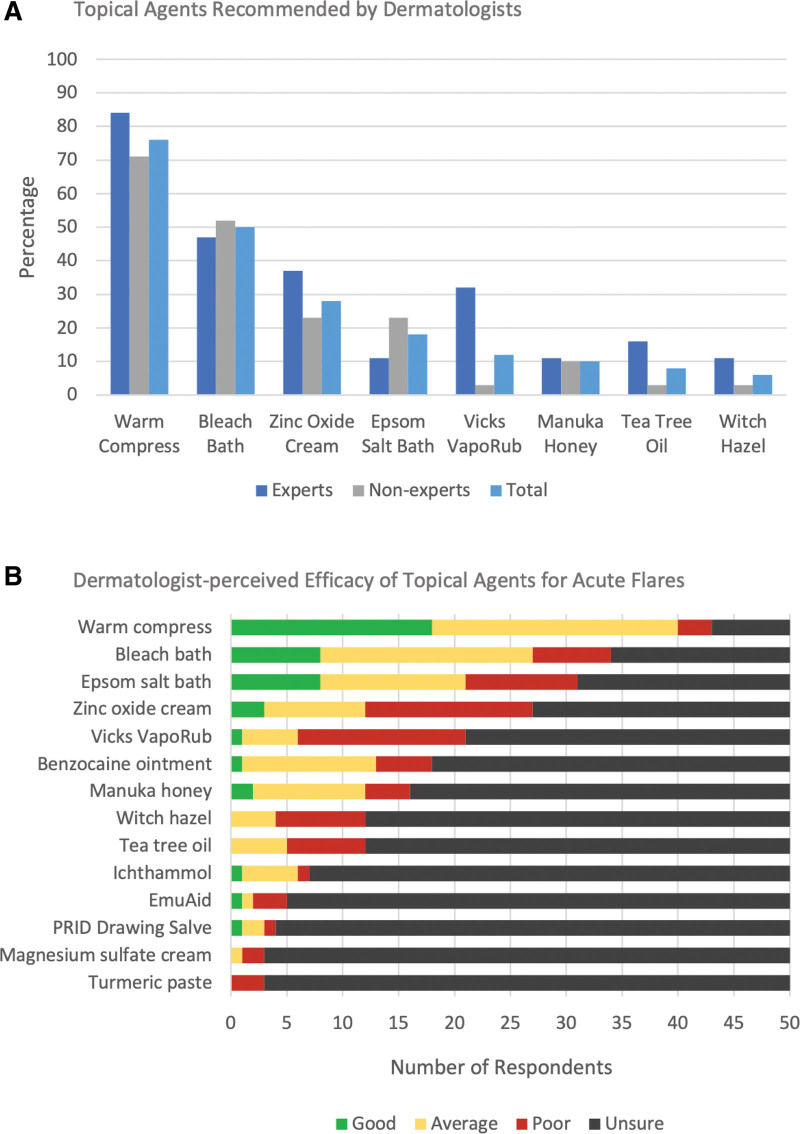
Perceptions of dermatologists on topical agents for acute hidradenitis suppurativa (HS) flares.

Over one-third of dermatologists surveyed did not counsel patients with HS about wound care dressings, highlighting the need to improve patient education. Only half of the respondents reported ordering insurance-covered wound care supplies for patients, which can help relieve financial burden for patients.^1^ There are numerous wound care supply companies across the United States that will distribute supplies to patients that are partly or completely covered by insurance. Typically, these orders require documentation of wound size and location in the clinical note and specific requests for supplies. Examples of commonly used supplies by patients with HS include abdominal pads, gauze dressing, and paper tape. Referrals to wound care can also be beneficial in increasing patient access to wound care supplies. Having access to a variety of dressings has been shown to improve patient quality of life by reducing pain, odor, and staining of clothes.^2^

Less than half of the dermatologists surveyed reported discussing clothing modifications, which can help reduce flares of HS.^3^ Wireless bras with thick bands, “boy shorts” or loose boxers for underwear, and loose-fitting clothing are recommended.^4^ HidraWear (HidraMed Solutions, Ireland) provides HS-specific clothing that was shown to improve comfort, mobility, and time spent on dressing changes.^5^ As HidraWear can be insurance-covered, dermatologists should consider this for patients with chronic drainage.

Study limitations include potential sampling and response bias due to the small sample size and low proportion of nonacademic dermatologists. Nevertheless, these findings underscore areas for improvement in wound care education for patients with HS.

## Conflicts of interest

J.M.R. has served on the advisory board for Novartis Pharmaceuticals, which is unrelated to this study. The remaining authors have no conflicts of interest to disclose.

## Funding

None.

## Study approval

This study was approved by the Northwestern University Institutional Review Board.

## Author contributions

SP and KS: Participated in writing, reviewing, and editing this article and analyzing data for this project. JMR: Participated in reviewing and editing this article and the conceptualization and administration of this project.

## Data availability

Data are available upon request from authors.

## Acknowledgments

We would like to thank the providers who participated in our study.

## Supplementary data

Supplementary material associated with this article can be found at http://links.lww.com/IJWD/A21.

## Supplementary Material


