# Supplementary material for: Wound care counseling of patients with hidradenitis suppurativa: perspectives of dermatologists
Source: Int J Womens Dermatol. 2023 Jul 18;9(3):e096. doi: 10.1097/JW9.0000000000000096 (PMC10353709; doi:10.1097/JW9.0000000000000096)
Supplement: Supplementary file 1 [file jw9-9-e096-s001.pdf]

Basic Information

How many years have you been practicing out of residency?

- ○ < 5 years
- ○ 5-10 years
- ○ 10-15 years
- ○ 15-20 years
- ○ 20+ years

What setting best describes your practice?

- ○ Academic
- ○ Multi-specialty group practice
- ○ Single specialty group practice
- ○ Solo private practice
- ○ Other

Please specify "other"

____

Where is your practice located?

- ○ Northeast (Connecticut, Maine, Massachusetts, New Hampshire, New Jersey, New York, Pennsylvania, Rhode Island and Vermont)
- ○ West (Arizona, California, Colorado, Idaho, Montana, New Mexico, Nevada, Oregon, Utah, Washington and Wyoming)
- ○ Midwest (Illinois, Indiana, Iowa, Kansas, Michigan, Minnesota, Missouri, Nebraska, North Dakota, Ohio, South Dakota and Wisconsin)
- ○ South (Alabama, Arkansas, Delaware, District of Columbia, Florida, Georgia, Kentucky, Louisiana, Maryland, Mississippi, North Carolina, Oklahoma, South Carolina, Tennessee, Texas, Virginia and West Virginia)
- ○ Noncontiguous states/territories (Alaska, Hawaii, Puerto Rico)
- ○ Other

Please specify "other"

____

What setting is your practice located?

- ○ Rural
- ○ Urban
- ○ Suburban

Do you consider yourself an HS expert?

- ○ Yes
- ○ No

What procedures do you perform for HS? Select all that apply.

- ☐ Intralesional steroid injections
- ☐ Deroofing
- ☐ Local excisions
- ☐ Laser hair removal
- ☐ Ablative laser
- ☐ None of the above

Wound Care Counseling & Recommendations

Do you counsel your HS patients on wound care management strategies?

- ○ Yes
- ○ No
- ○ Sometimes

What topics do you regularly discuss with your patients? Select all that apply.

- ☐ Types of wound care dressings for daily wound care
- ☐ Management of inflamed nodules or abscesses
- ☐ Clothing choices
- ☐ When to call the office or go to the emergency department
- ☐ Dangers of self-lancing or squeezing abscesses
- ☐ Other

Please specific "other"

____

Do you feel that patients with HS are well-educated about wound care dressings and strategies?

- ○ Yes
- ○ No

How many minutes on average do you spend per visit counseling on wound care for HS patients?

- ○ < 2 minutes
- ○ 2-5 minutes
- ○ > 5 minutes

Do you feel that patients should get more education and counseling on wound care dressings and at-home management of acute nodules?

- ○ Yes
- ○ No

Do you order wound care supplies for HS patients?

- ○ Yes
- ○ No
- ○ Sometimes

Which wound care supplies do you typically prescribe?

____

Which of the following do you recommend to your patients for daily drainage and symptoms? Select all that apply.

- ☐ Tissues or toilet paper
- ☐ Gauze dressing
- ☐ Abdominal pads
- ☐ Antispetic dressing
- ☐ Panty liners/sanitary pads
- ☐ Adult diapers
- ☐ Other:
- ☐ I do not counsel on wound care dressings

Please specify "other"

____

Do you counsel your HS patients on clothing choices or modifications?

- ○ All my patients
- ○ Some of my patients
- ○ None of my patients

What specific clothing products or modifications do you counsel HS patients about? Select all that apply.

- ☐ Boxer shorts / Boy shorts
- ☐ Wireless bras
- ☐ Seamless pants
- ☐ Bra liner
- ☐ Abdominal liner
- ☐ Hidrawear crop top or similar
- ☐ Other:

Please specify "other"

____

Do you discuss any of the following products with your HS patients for at-home management of acute nodules?

- ☐ Manuka honey
- ☐ Vick's VapoRub
- ☐ Warm compress
- ☐ Epsom salt bath
- ☐ Bleach or chlorine bath
- ☐ PRID Homeopathic Drawing Salve
- ☐ Ichthammol
- ☐ Boil Ease or benzocaine ointment
- ☐ Desitin or diaper rash paste (zinc oxide)
- ☐ Magnesium sulfate paste
- ☐ EmuAid
- ☐ Witch hazel
- ☐ Tea tree oil
- ☐ Turmeric Paste
- ☐ Other
- ☐ None of the above

Please specify "other"

____

How effective do you think the following are for at-home management of acute HS nodules?

	1 = Poor	2	3 = Average	4	5 = Excellent	Do not know
Manuka Honey	○	○	○	○	○	○
Vick's VapoRub	○	○	○	○	○	○
Warm compress	○	○	○	○	○	○
Epsom salt bath	○	○	○	○	○	○
PRID Homeopathic Drawing	○	○	○	○	○	○
Ichthammol	○	○	○	○	○	○
Boil Ease or benzocaine	○	○	○	○	○	○
Magnesium sulfate paste	○	○	○	○	○	○
Diaper rash paste (zinc oxide)	○	○	○	○	○	○
EmuAid	○	○	○	○	○	○
Witch Hazel	○	○	○	○	○	○
Tea tree oil	○	○	○	○	○	○
Bleach/chlorine bath	○	○	○	○	○	○
Turmeric paste	○	○	○	○	○	○
